# Correction and calibration of atmospheric impact observations in GOES GLM data

**DOI:** 10.1111/maps.13926

**Published:** 2022-11-24

**Authors:** Robert L. Morris, Jeffrey Claiborne Smith, Jessie L. Dotson, Eric C. Stern, Randolph S. Longenbaugh

**Affiliations:** ^1^ SETI Institute 339 Bernardo Ave, Suite 200 Mountain View California 94043 USA; ^2^ NASA Ames Research Center Moffett Field California 94035 USA; ^3^ Sandia National Laboratories Albuquerque New Mexico 87185 USA

## Abstract

The Earth's atmosphere is impacted daily by both meteoroids and artificial objects. Calibrated observations of the emitted light at sufficiently high sampling rates can enable or improve the estimation of impactor attributes such as size, cohesion, trajectory, and composition, but are difficult to obtain owing to the unpredictability, brevity, and high dynamic (brightness) range of impacts. Ground‐based camera systems have successfully monitored small regions of the atmosphere at video frame rates and with limited radiometric capabilities, but most impacts occur over the 70% of the Earth's surface covered by water and are therefore missed by these networks. The Geostationary Lightning Mapper (GLM) instruments aboard Geostationary Operational Environmental Satellites 16 and 17 provide near‐hemispherical coverage at 500 frames per second. These data have been shown to contain the signatures of many independently confirmed impacts, often from both viewing angles simultaneously, and constitute an observational resource that is currently unparalleled in the public domain. NASA's Asteroid Threat Assessment Project has implemented an automated impact detection pipeline that processes data from GLM daily. Given a detected impact, the GLM data contain a wealth of information for use in quantitative follow‐up analyses. However, impact events differ from lightning in ways that violate key assumptions built into GLM's design. The result is that GLM's onboard processing introduces errors into pixel observations of impact events and the calibrated energies near the periphery of the detector may be substantially overestimated. We present methods for mitigating these and other issues to produce a data product more suitable for impact analyses than the existing GLM lightning product.

## Introduction

Increasing the number and quality of impact light curves available for study stands to benefit fields ranging from planetary defense to solar system formation. Data from the Geostationary Lightning Mapper (GLM) instruments aboard Geostationary Operational Environmental Satellites (GOES) 16 and 17 have been shown to contain observations of many impact events in a region covering nearly half of the Earth's surface (Jenniskens et al., 2018). Calibrated observations at the sampling rate and dynamic range of GLM have the potential to advance our understanding of impactors and their interactions with the Earth's atmosphere. Other publicly available sources of calibrated observations are primarily ground‐based camera networks, many of which are members of the Global Fireball Observatory (GFO) (Devillepoix et al., 2020), and include the European Fireball Network (Flohrer et al., 2012), Desert Fireball Network (https://dfn.gfo.rocks), NASA Meteorite Tracking and Recovery Network (http://fireball.seti.org), NASA All Sky Fireball Network (https://fireballs.ndc.nasa.gov), Sky Sentinel (http://goskysentinel.com), Cameras for Allsky Meteor Surveillance (CAMS) (Jenniskens et al., 2011), Spanish Fireball Network (Trigo‐Rodríguez et al., 2006), and Fireball Recovery and Interplanetary Observation Network (https://www.fripon.org). These networks provide limited coverage, frame rates, and radiometric capabilities compared to GLM. Nodes in the GFO cover less than 2% of the Earth's surface and share a design heritage with the Desert Fireball Network, which is based on a consumer digital camera (Howie et al., 2017). These cameras operate at frame rates no higher than about 30 Hz and though some are now equipped with add‐on radiometers (Buchan et al., 2018), most are not. For the fireballs they observe, these networks may enable better trajectory estimates than GLM (Peña‐Asensio et al., 2021), but not better radiometry. Because they observe impacts from beneath local weather conditions and through highly direction‐dependent air columns, the measurements they produce are necessarily far less uniform than those from GLM's orbit. Prior to GLM, the only sources of similar broad‐field, space‐based observations were U.S. Government Department of Defense satellites (USG satellites) with prohibitive restrictions on access to data and instrument specifications (Bouquet et al., 2014; Tagliaferri et al., 1998). GLM's combination of data quality, transparency, and near‐real‐time availability (data product latency is less than 20 s) are currently unparalleled, motivating this work.

### GLM Instrument and Data Products

Each of the GLM instruments images nearly a third of the Earth's surface every 2 ms at 8–14 km per pixel spatial resolution. The 1372 × 1300 pixel charge‐coupled device (CCD) array incorporates a unique variable‐pitch layout intended to image approximately equal surface areas with each pixel. The nominal 1.1 nm pass band centered at 777.4 nm is designed to capture characteristic lightning emissions and optimize both detection and energy measurement in the face of varying solar illumination (Goodman et al., 2013).

The high frame rate results in a data stream far exceeding the nominal 7.7 Mbps downlink rate, which precludes the return of all data collected by the instrument. An onboard detection algorithm is therefore executed by several real‐time event processors (RTEPs) to identify potential lightning as impulsive pixel events (the basic unit of data from the instrument), which are recorded and downlinked. A pixel event is defined as a measurement exceeding ∼4 times the noise level above the estimated background. Background estimates at each pixel and frame are calculated as a running average of the current pixel measurement and previous background estimate. A 14‐bit full‐image snapshot of the background estimates is recorded and downlinked every 2.5 min.

The downlinked data for a single event consist of the 14‐bit, background‐subtracted event amplitude, the five most significant bits (MSBs) of the background estimate, and integers identifying the pixel and frame on which the event occurred. GLM data products are categorized as levels 0, 1, and 2, corresponding to raw data from spacecraft, pixel‐level processing, and group and flash processing, respectively. Level‐0 (L0) data are downlinked and undergo ground processing to produce the Level‐2 (L2) data product intended for public consumption. The L2 event data are filtered to remove false positives (events unlikely to have been caused by lightning), corrected for overshoot and crosstalk artifacts, calibrated, and navigated to geodetic coordinates. They are then organized into groups and flashes, which are meaningful to lightning researchers. GLM data files are delivered in the netCDF‐4 file format, with each L0 file containing data from a 5‐min time window and each L2 file spanning 20 s.

### ATAP Impact Detection

NASA's Asteroid Threat Assessment Project (ATAP) has designed and implemented a bolide detection pipeline (Smith et al., 2021), which processes GLM L2 data on a daily basis and automatically identifies likely bolide signatures. Note that, although the objective is to detect small asteroid impacts, the algorithm cannot currently distinguish these from reentries of artificial objects. The detection algorithm operates on L2 group data and broadly consists of three steps: clustering, feature extraction, and classification. A hierarchical clustering method is applied to the latitude, longitude, and time coordinates of GLM groups. Features are then extracted from each cluster, and the resulting feature vectors are classified using a random forest (a machine learning method based on decision trees) trained on data vetted by a human expert. Each feature vector is assigned a measure of confidence that it represents a bolide and the results are written to a file along with a series of diagnostic figures. Detection results are vetted by a human expert and likely bolides are published on https://neo‐bolide.ndc.nasa.gov. While the pipeline's detection performance is quite good, with nearly 88% of detections confirmed by the human expert (Smith et al., 2021), it does not necessarily identify the complete subset of GLM pixel events triggered by the impact. In fact, it is common for a single impact to result in several detections, each triggered by a different subset of the data.

### A Derivative Data Product for Impact Analysis

While much can be learned from the detection statistics alone, there is a wealth of valuable information in the pixel data associated with a given detection. These data contain high time‐resolution information about the energy released during the impact and, to a lesser extent, the location of the impactor. A near‐term goal of the ATAP is to use these data to produce calibrated light curves (radiant flux or energy as a function of time) for each detected object. Fitting existing models of bolide fragmentation, energy deposition, and luminous efficiency to observed light curves can help validate the models or illuminate their shortcomings. More and better light curves will also facilitate better inferences about object attributes such as diameter, density, and aerodynamic strength (Peña‐Asensio et al., 2022; Tarano et al., 2019; Trigo‐Rodríguez & Llorca, 2006; Wheeler et al., 2017). Before we can hope to produce accurate light curves, we must first extract the relevant data points, correct systematic errors, and recalibrate the data (the subject of this paper). Ideally, for a given impact event, we would like to know (1) the locations of all GLM pixel events (pixel and frame ID numbers) triggered by the impact, (2) the full 14‐bit pixel value associated with each event along with its observational uncertainty, (3) the fraction of the total pixel value that is due to background illumination, and (4) a calibration model that maps each pixel's background‐subtracted value to radiant energy in the nominal pass band. The remaining sections describe the methods by which we estimate or bound these quantities and manage sources of uncertainty, with the goal of maximizing the utility of GLM data for impact event research.

Our methods overcome a number of challenges posed by the unique characteristics of GLM data products. First, GLM's onboard processing algorithms, which were designed for lightning observation, tend to be destructive when applied to impact events, either partially or entirely discarding pixel observations. Second, the calibration tables available for lightning observations are poorly suited to impact observations, especially near the periphery of the detector. Finally, the L2 lightning data products do not contain all the information necessary to accurately derive the calibrated flux of an impact event. Information from the raw L0 GLM data stream must be incorporated as well. Unfortunately, the L2 and L0 data are not easily reconciled. In the following sections, we describe in detail our methods for addressing these issues. The Data Extraction section addresses the problems of extracting the subset of pixel observations associated with an impact and combining the pertinent information from raw and processed GLM data products. The Data Correction and Augmentation and Calibration sections describe, respectively, our efforts to mitigate the destructive effects of onboard processing and produce a calibration model appropriate for a given impact event.

## Data Extraction

For each detected impact, the ATAP detection pipeline records the list of L2 pixel events that triggered the detection and the names of the data files containing them. We use these events as seed points and perform cluster analysis to identify additional pixel events that were likely triggered by the impact. Next, we identify corresponding L0 event data and extract pixel location, uncalibrated event amplitude, and 5‐bit background estimates for each event. Finally, we combine the L0 and L2 event data. To do so, we must identify corresponding events in the L0 and L2 data sets. L0 events are uniquely identified by their integer pixel (CCD) coordinates and time according to the system clock. L2 events are identified by their navigated surface coordinates in latitude, longitude, and time (accounting for light travel). Unfortunately, because the L2 event processing does not preserve the pixel coordinates or spacecraft times, we are forced to infer the correspondence by registering L0 and L2 point clouds. The flow of data through these steps is shown in Fig. 1. The Event Clustering, Image Navigation, and Registration sections discuss each step in more detail. It is worth noting that merging the L0 and L2 data is only necessary to get the benefits of the GLM ground processing described previously in the GLM Instrument and Data Products section. If one were to fully replicate the navigation and false event filtering algorithms, it would be possible to work with the L0 data alone.

**Fig. 1:**
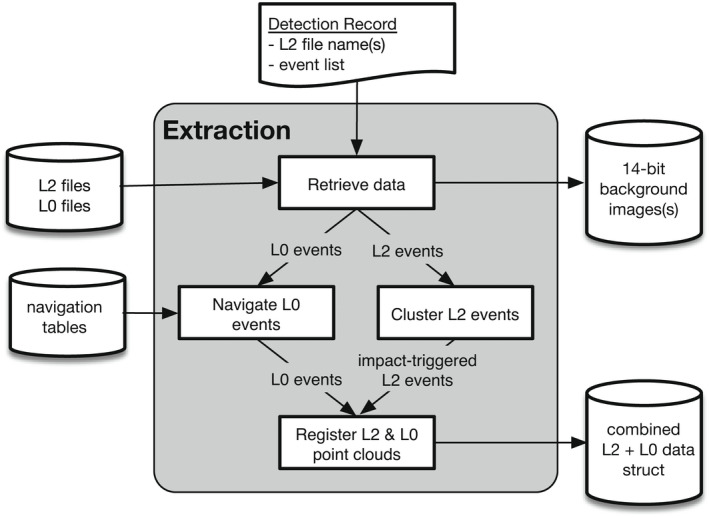
Block diagram of the data extraction process. A detection record containing L2 file names and detected event IDs is submitted to the extraction module, which retrieves the appropriate L2 and L0 files, applies clustering to identify L2 events likely triggered by the impact, maps L0 events to approximate lat/lon coordinates, and registers the L2 and L0 point clouds. The primary output is a structure containing information from both L0 and L2 files for each event associated with the impact. Optional outputs include the 14‐bit background image(s) obtained at 2‐min intervals and the image's trajectory. Note that here the term “trajectory” refers not to the path of the impactor in space, but instead to the linear path of the impactor's image on the CCD.

### Event Clustering

Given the set of L2 events delivered by the ATAP bolide detector, we examine the local neighborhood more closely and include additional events that may be associated with the impact. We start by identifying a plausible neighborhood in space and time and pruning any events outside of it. Each remaining event is characterized by its latitude, longitude, and time coordinates $p=\left(\phi ,\theta ,t\right)$ and its energy *w*. We assume for convenience that impact‐triggered event coordinates approximately follow a multivariate normal distribution with a prominent major axis and comprise the dominant cluster in the neighborhood. Under this assumption, we iteratively compute the weighted mean and covariance matrix of the data points and discard the most outlying 1% until some minimum fraction (currently 20%) of points remains.

On the *i*th iteration, let **P** denote the *N*‐by‐3 matrix of row vectors representing the remaining centered points $p_{n}\;-\;u_{i}$, where *n* = 1, 2, … , *N*, and let $W=\mathrm{diag}\left(w_{1},w_{2},\ldots ,w_{N}\right)$ denote the diagonal matrix of weights. The weighted mean vector and covariance matrix for the *i*th iteration are given by

(1) $$u_{i}=\frac{1}{\sum w_{n}}\sum w_{n}p_{n},$$

and

(2) $$C_{i}=\frac{1}{\sum w_{n}}\sum P^{T}\mathrm{WP},$$

respectively. On each iteration, we compute the Mahalanobis distance (the number of standard deviations from the mean) (Mahalanobis, 1936) of each remaining point from the distribution $N_{i}=N\left(u_{i},C_{i}\right)$. The most distant 1% are discarded, and the procedure is repeated until 20% of the original points remain. At this point, we analyze the behavior of the distributions $N_{i}$ across iterations. In doing so we borrow the concept of a median vector (Liu, 2013) to define the median distribution in the set $N\;=\left\{N_{1},N_{2},\ldots ,N_{I}\right\}$, where *I* is the number of iterations, as the distribution that minimizes the sum of Bhattacharya distances (measures of similarity between pairs of distributions; Bhattacharyya, 1943) to all others in the set:

(3) $$\bar{N}=\underset{N_{n}\;\in \;N}{\arg\;\min}\sum_{i=1}^{N}D_{\text{Bhat}}\left(N_{n},N_{i}\right).$$

Intuitively, $\bar{N}$ represents the most stable point during the iterative process. Finally, we discard events whose Mahalanobis distance is greater than *k* standard deviations from $\bar{N}$, where *k* is arbitrarily set to 10 in the current implementation. We also include an optional bias factor, *B*, in the range [0,1] that increases the distance metric for distributions computed from fewer points. The multiplicative bias applied to the *i*th distance is given by *b*(*i*) = 1 − *B* + *B*(*i* − 1)∕(*I* − 1) because many of the $N_{i}$ may produce similar metrics and, all else being equal, we prefer distributions estimated from greater numbers of points. The right panel of Fig. 2 shows the sums of distances from Equation 3 derived from the point cloud of a recent bolide in the left panel. The minimum value corresponds to the optimal cluster.

**Fig. 2:**
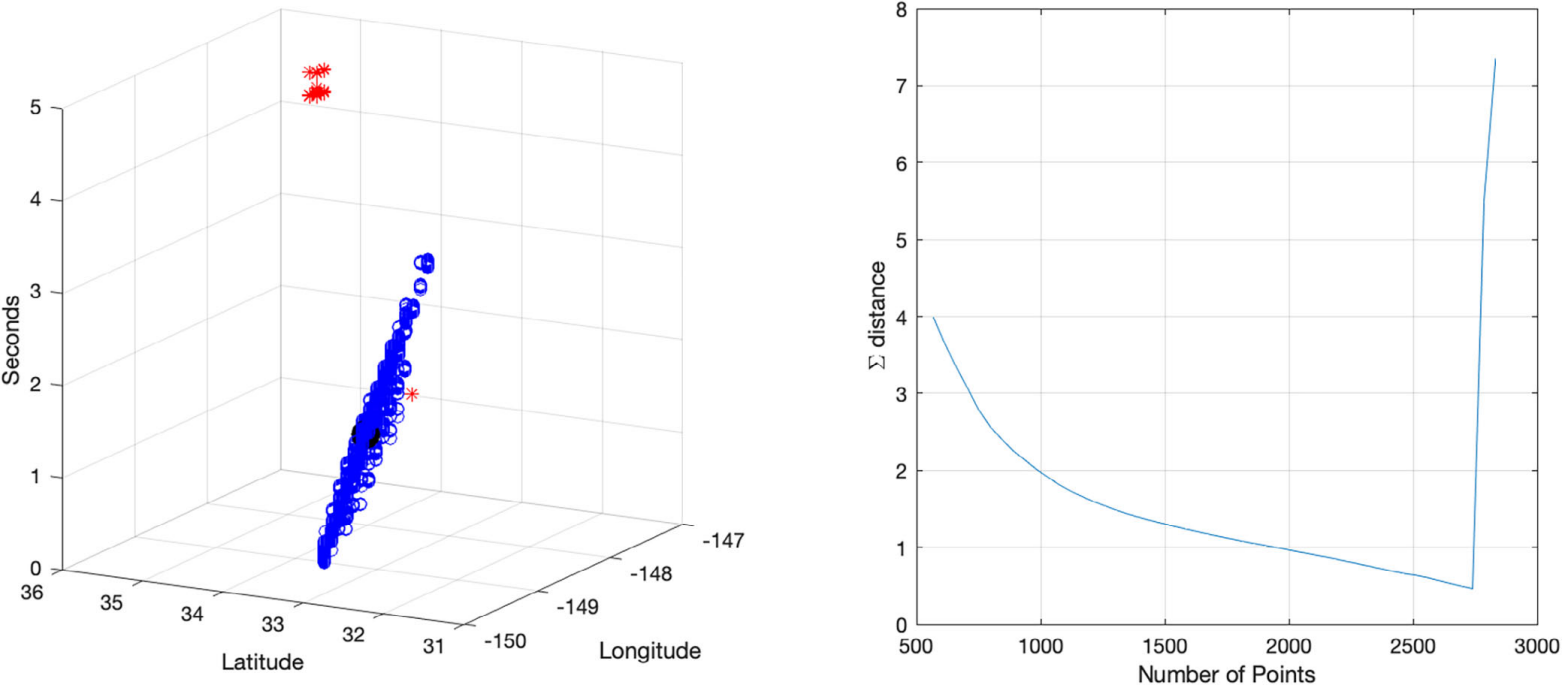
Clustering results for a large bolide over the Pacific on September 10, 2022. The left panel shows event locations in ϕ, θ, *t* with discarded points in red and the cluster mean in solid black, while the right shows the sum of distances from each hypothetical distribution to all the others as a function of the number of points used to estimate it. The most distant points are eliminated in each iteration; the first iteration corresponds to about 2800 points and the last to just over 500. The precipitous drop in the sum of distances indicated on the far right corresponds to elimination of the obvious outliers during the initial few iterations. (Color figure can be viewed at wileyonlinelibrary.com.)

We also optionally identify the line segment in ϕ, θ, *t* that best describes the path of the image centroid by performing standard principal component analysis on the weighted covariance matrix $\bar{C}$ to obtain the principal axis. The endpoints are then determined by projecting each centered point onto the axis and identifying the extremes in either direction.

### Image Navigation

The L2 processing maps, or navigates, the CCD coordinates of each pixel event to geodetic coordinates corresponding to the center of the pixel footprint at the time of the event. We have implemented all aspects of the image navigation procedure used in GLM ground processing (van Bezooijen et al., 2016) except for accounting for the Earth's nutation. Annual nutation effects are typically a few tens of arc seconds, which is comparable to the field of view of a single GLM pixel (~21 arcsec at the center of the focal plane). This virtually guarantees the success of the registration algorithm described in the Registration section. Since our goal is to identify corresponding L0 and L2 events, we navigate the L0 events to an ellipsoid approximating the tropopause as in the L2 ground processing. Navigation of GLM events can be boiled down to the following three steps:

1. Compute a unit vector $v_{\mathrm{gf}}$ along the line of sight in the GLM functional coordinate frame (defined in van Bezooijen et al., 2016).
2. Rotate $v_{\mathrm{gf}}$ into the International Terrestrial Reference System to obtain the vector $v_{\mathrm{it}}$.
3. Calculate the geodetic latitude and longitude of the point at which the line along $v_{\mathrm{it}}$ intersects the lightning ellipsoid.

Because the navigation procedure is involved and relies on data from external sources, we chose to create approximate lookup tables as a matter of convenience. A year of data from the GOES 16 GLM were navigated, and statistics on the navigated positions and light propagation times were collected and stored in a lookup table. The same was done for GOES 17 GLM data in both yaw flip states. The lookup tables provide an approximation sufficiently close for point cloud registration, as described in the Registration section to resolve any discrepancies.

### Registration

To combine the pertinent L0 and L2 data, we identify corresponding pixel events in the two data products by way of point cloud registration. The navigation of L2 events by the GLM ground processing pipeline produces a latitude ϕ, longitude θ, and time stamp *t* for each event $p_{m}={\left\langle \phi ,\theta ,t\right\rangle }^{T}$, where *m* = 1, 2, …, *M* indexes the set of pixel events triggered by the impact. The approximate navigation lookup tables described in the previous section are used to map all L0 events to approximate coordinates $q={\left\langle \hat{\phi },\hat{\theta },\hat{t}\right\rangle }^{T}$. In most cases, the approximation is close enough (within a few pixels or tens of arcsec) that we can isolate the corresponding L0 events in a reasonably small set (typically about twice the number of events as in the L2 set). We then apply an iterative closest point (ICP) algorithm (Wilm, 2012) to register the L2 and L0 point clouds, an example of which is shown in Fig. 3. The ICP algorithm returns an affine transformation that can be applied locally to correct the approximate L0 coordinates. A one‐to‐one mapping between each L2 point and its closest (corrected) L0 point is then established. In more detail, the procedure is as follows:

1. Navigate L0 events using the appropriate lookup table.
2. Prune L0 events outside the latitude, longitude, and time boxes bounding the L2 data, allowing for user‐defined tolerances.
3. Stretch the data along the time and longitude dimensions so that points representing pixel observations lie on an approximately uniform grid in three dimensions.
4. Apply the ICP algorithm to determine the affine transformation that best registers L0 points with L2.
5. For each L2 point, identify the closest registered L0 point and remove the pair from circulation, ensuring a one‐to‐one mapping between L0 and L2 events. The mean distance between correctly registered point sets should be close to zero.

**Fig. 3:**
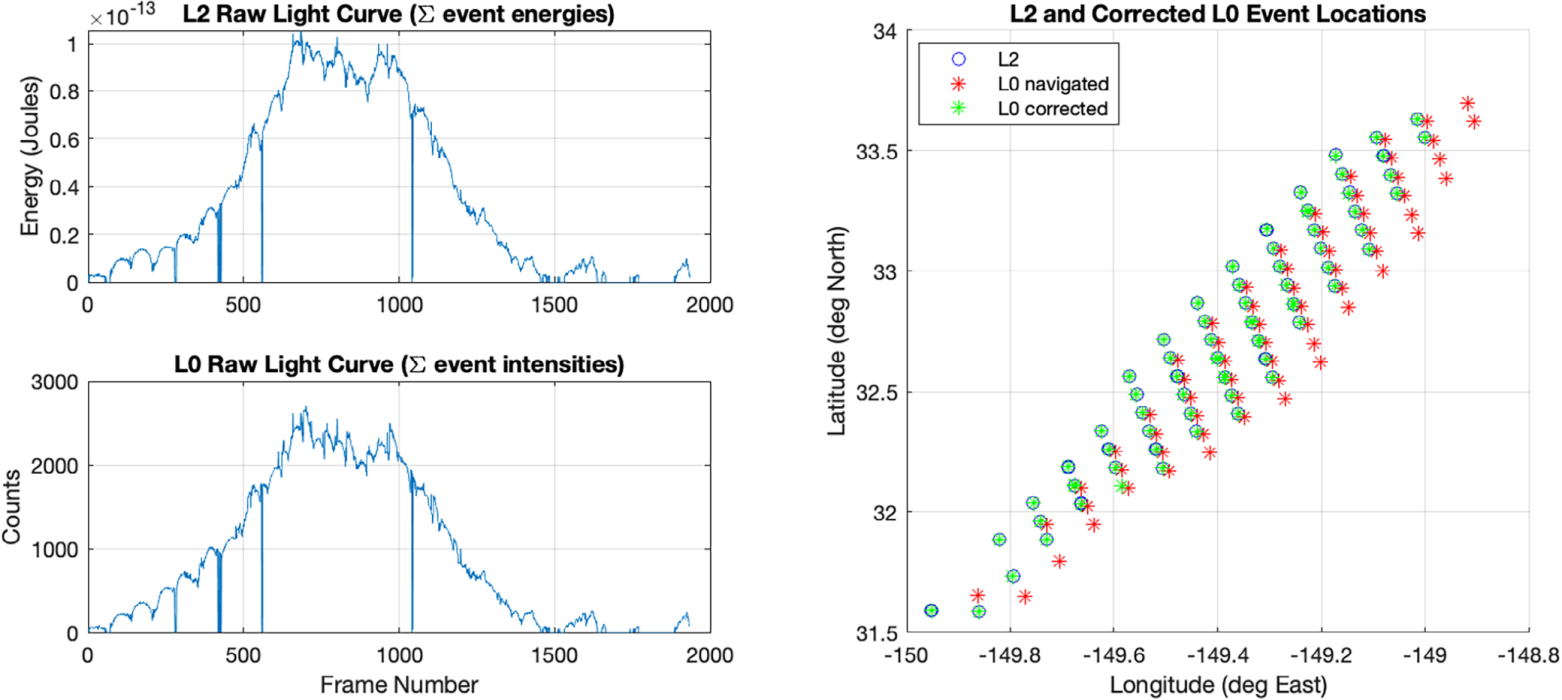
Results of L0/L2 event registration on data from the 9/10/2020 bolide. The left panels show good qualitative agreement between the light curves formed by summing matching events on each frame. The right panel illustrates the errors in our approximate navigation and the corrective effect of the affine transformation on L0 spatial coordinates (time coordinates were omitted for clarity). (Color figure can be viewed at wileyonlinelibrary.com.)

Frame times (s) are scaled in step 3 by a factor of 24 to yield roughly uniform point spacing in all three dimensions, based on the average spatial distance between pixel centers (roughly 0.1°). Doing this helps with the version of ICP we are currently using because it relies on a Euclidean distance metric.

A combined L2 + L0 data structure, which contains only events present in both L2 and L0, is returned along with the affine transformation. The transformation can be used to correct the geodetic and time coordinates of additional L0 events, if desired.

A few details about the affine transformation are worth mentioning. The ICP algorithm returns a transformation matrix $A_{\mathrm{ICP}}$, which can be thought of as a product of rotation, scaling, and skew matrices, along with a translation vector $T_{\mathrm{ICP}}$. Because these pertain to data that have been scaled along the time and longitude axes, as described in step 3 above, they cannot be directly applied to correct the unscaled L0 data. Let $S=\mathrm{diag}\left(1,\cos{\left(\bar{\phi }\right)}^{-1},24\right)$ denote the 3 *×* 3 diagonal matrix that performs the scaling of vectors, where $\bar{\phi }$ represents the mean longitude of the impact. The rotation matrix **A** and translation **T** that apply to unscaled data are then given by $A=S^{-1}A_{\mathrm{ICP}}S$ and $T=S^{-1}T_{\mathrm{ICP}}S$. The corrected coordinates $q^{\prime }$ for a table‐based approximation **q** are given by

(4) $$q^{\prime }=\mathrm{Aq}+T.$$

## Data Correction and Augmentation

The GLM instrument and its onboard event processing algorithms are well suited for observing lightning activity. But because impact events do not resemble lighting, they violate key assumptions built into the GLM system. As a result, impact observations will contain systematic errors, an example of which is shown in Fig. 4. In this example, we see how the adaptive background algorithm attempts to follow the increasing brightness trend of an impact. Because the lowest nine bits of each background estimate are discarded to reduce data volume, some of the impact signal is discarded along with it. As the impact's brightness begins to decrease, the adaptive background will overtake it at some point and subsequent observations will be discarded entirely. While not a problem when measuring impulsive lightning strikes, for impact events of increasing duration, it introduces likewise increasing errors. Unfortunately, larger impacts are usually the most interesting. Fortunately, these errors can at least be bounded and in many cases corrected entirely by leveraging our knowledge of the onboard algorithms. For reasons that will become clear in the 14‐Bit Background Reconstruction and Bounding section, such errors can usually be more completely corrected in cases of larger impacts. In this section, we describe the procedure by which we reconstruct or bound the 14‐bit pixel values.

**Fig. 4:**
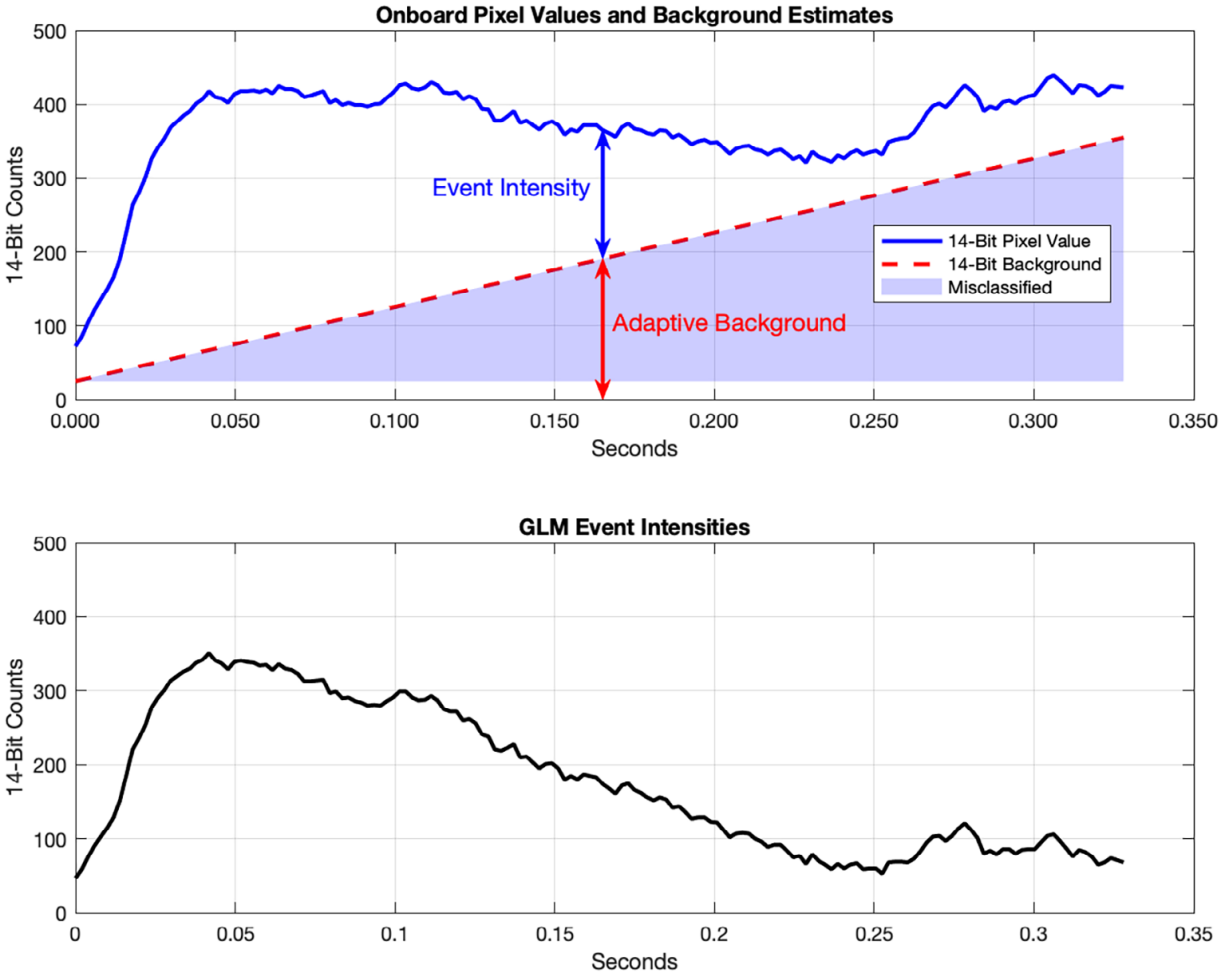
An example of the effects of onboard background subtraction on a pixel from GOES‐17 observations of a bolide on February 22, 2022 (11:21 UTC at 33.4°S and 122.1°W). The adaptive onboard background estimate will creep upward in response to a sustained signal, misclassifying more and more bolide signal as background. Because of the misclassification, the returned L0 event intensities (bottom) do not accurately reflect the relative magnitudes of the radiant flux from the bolide. In this example, it was possible to exactly reconstruct the 14‐bit onboard values (top panel) for all of the returned pixel events. (Color figure can be viewed at wileyonlinelibrary.com.)

### GLM False Event Filters

In order to identify and reject likely false lightning events and also to correct for overshoot and crosstalk artifacts, a number of false event filters (FEFs) are applied to L0 events in the ground processing when producing the L2 data product. In our experience, the vast majority of identified false lighting events are due to systematic errors and not to impact‐related flux that we would want to preserve. For this reason, we do not try to incorporate additional L0 event data that do not have corresponding events in L2. There are, however, two FEFs—the overshoot and crosstalk filters—that can modify the L0 event intensity values without rejecting the events (Edgington et al., 2019). As illustrated in Fig. 5, we compute additive corrections for overshoot and crosstalk and apply them to the results of the 14‐Bit Background Reconstruction and Bounding section. Barring any unintended algorithmic discrepancies, these corrections should be identical to those applied during GLM ground processing.

**Fig. 5:**
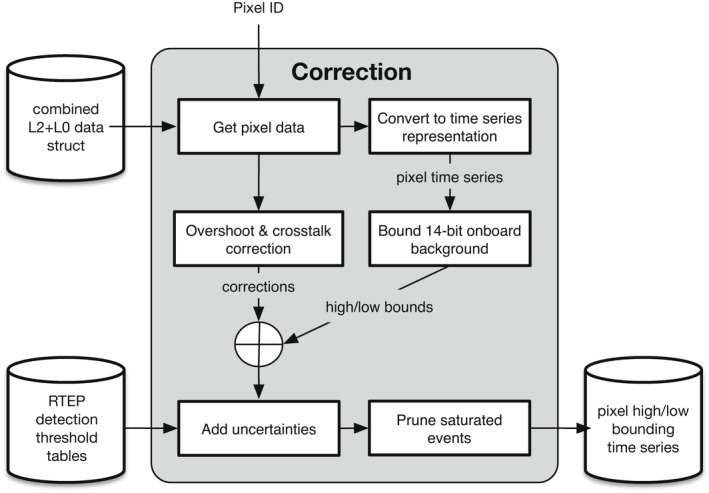
Block diagram of the procedure to mitigate artifacts in the extracted impact data. Given a pixel ID, data from the specified pixel are extracted from the combined L2 + L0 structure. The data are converted to time‐series representation and upper and lower bounds on the 14‐bit onboard pixel values are computed. Additive overshoot and crosstalk corrections are computed in parallel and applied to the bounding time series. Finally, uncertainties are derived from the real‐time event processor (RTEP) threshold tables, and saturating observations are optionally removed.

### 14‐Bit Background Reconstruction and Bounding

In most cases, a bolide observed by GLM will cause rapidly rising background estimates in pixels where the bolide flux is concentrated, allowing 14‐bit background values to be at least partly reconstructed. At a minimum, we can identify upper and lower bounds on the 14‐bit values, and our knowledge of the system often allows them to be narrowly constrained.

The event intensity *e*_*k*_ = *d*_*k*_ − *b*_*k*_ is defined as the difference between the measured value and the 14‐bit background estimate (Goodman et al., 2012). The background level for frame *k* is estimated by the weighted sum of the current measurement *d*_*k*_ and the previous background level *b*_(*k*−1)_ (Benz et al., 2018),

(5) $$b_{k}={\mathrm{Md}}_{k}+\left(1\;-\;M\right)b_{\left(k-1\right)},$$

where the value of *M* for both GLM instruments is 1/16. The change in background level between successive frames is restricted, or clamped, such that it never lies outside the range [δ_min_, δ_max_]. Values less than δ_min_ are set to δ_min_ and values greater than δ_max_ are set to δ_max_. Since mid‐2018, the clamp values for GOES 16 have been ±2. The values for the GOES 17 GLM were +2∕−4 until October 15, 2019 when they were changed to ±4 (C. Tillier, private communication, December 11, 2019).

Given a string of successive events, we can infer the 14‐bit background values for all events if at least one is known. In the case where *b*_(*k*−1)_ is known and there is no clamping applied, the backward‐looking change in background level is given by

(6) $$\Delta^{-}=b_{k}\;-\;b_{\left(k-1\right)}=\frac{M}{\left(1\;-\;M\right)}e_{k}.$$

With clamping applied at a maximum change in background of δ_max_ and a minimum change of δ_min_, the value of *b*_*k*_ is

(7) $$b_{k}=\left\{\begin{array}{ll} b_{k-1}+\delta_{\max}, & \text{if}\;\Delta^{-}>\delta_{\max}\\ b_{k-1}+\delta_{\min}, & \text{if}\;\Delta^{-}<\delta_{\min}\\ b_{k-1}+\Delta^{-}, & \text{otherwise} \end{array}\right..$$

Similarly, if *b*_(*k*+1)_ is known, then the forward‐looking change is given by

(8) $$\Delta^{+}=b_{k}\;-\;b_{\left(k+1\right)}=\frac{-M}{\left(1\;-\;M\right)}e_{k},$$

and *b*_*k*_ by

(9) $$b_{k}=\left\{\begin{array}{ll} b_{k+1}\;-\;\delta_{\min}, & \text{if}\;\Delta^{+}>-\delta_{\min}\\ b_{k+1}\;-\;\delta_{\max}, & \text{if}\;\Delta^{+}<-\delta_{\max}\\ b_{k+1}+\Delta^{+}, & \text{otherwise} \end{array}\right..$$

To reconstruct the 14‐bit background signal as well as possible, we do the following for each pixel:

Step 1. Identify known 14‐bit background values by locating changes in the most significant five bits between successive frames. If the step is upward, use the post‐step value. If it is downward, use the pre‐step value. To clarify, this step is based on reasoning that whenever the lowest bit in the 5‐bit background changes its value, we know at that instant that the 14‐bit binary value onboard was either changing from xxxx0111111111 to xxxx1000000000 or from xxxx1111111111 to xxxx0000000000 (“x” is used here to denote a placeholder value that may be either “0” or “1”).

Step 2. Use Equations (6), (7), (8), (9) to calculate neighboring background values wherever possible. If all events are contiguous and there is at least one change in the 5‐bit background value, we can exactly reconstruct the 14‐bit background (shown in green in Fig. 6).

**Fig. 6:**
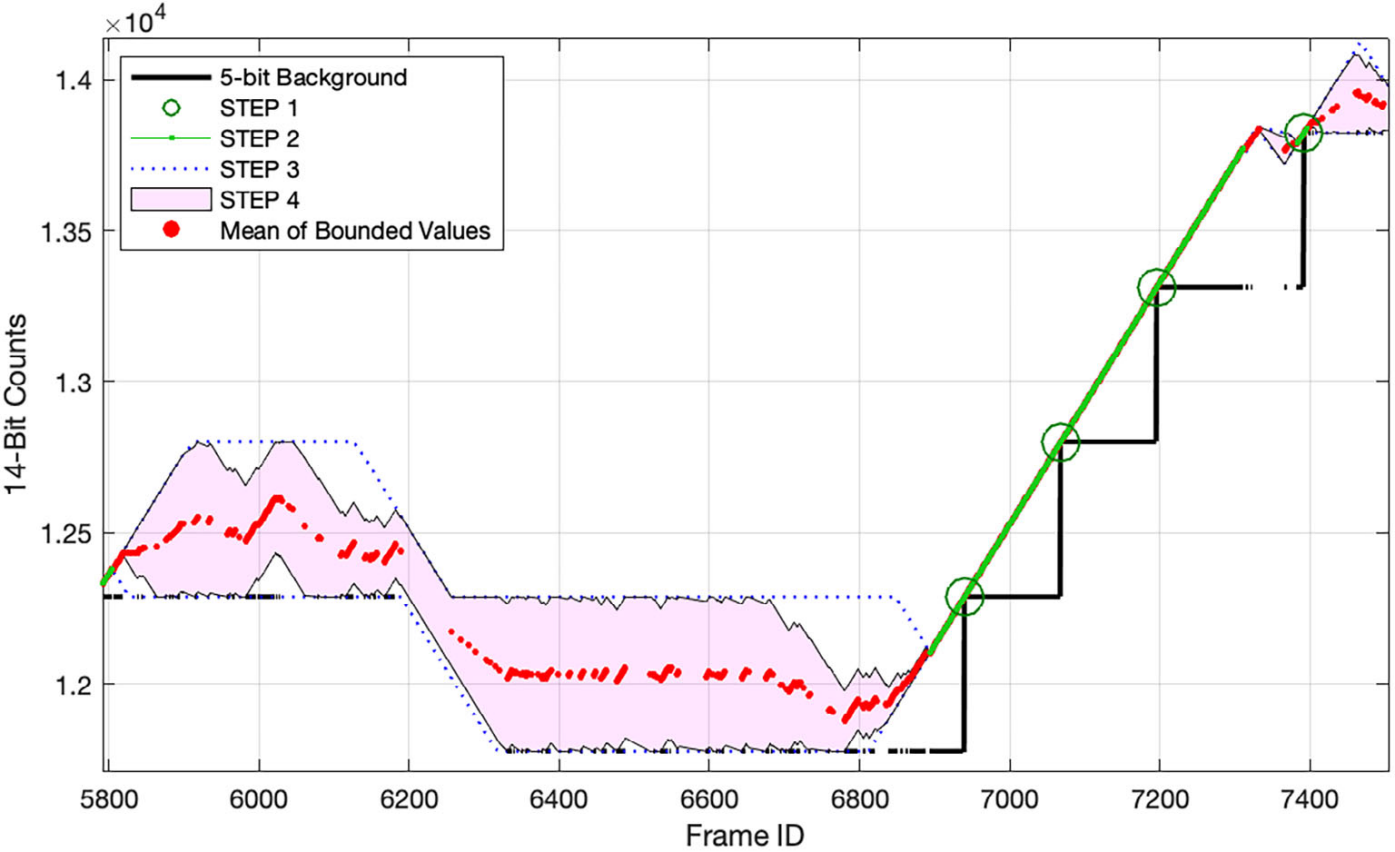
Illustration of steps in the 14‐bit background reconstruction and bounding process. The 14‐bit values are known on frames in which the 5‐bit background values change (STEP 1). Adjacent 14‐bit values can be exactly inferred (STEP 2). Nonadjacent values can be bounded (STEPS 3 and 4). (Color figure can be viewed at wileyonlinelibrary.com.)

Step 3. For frames on which 14‐bit background values cannot be reconstructed exactly, determine upper and lower bounding curves assuming no information beyond any reconstructed 14‐bit values from other frames and the clamping limit. This situation arises when (a) there is no change in the top five bits for the entire time series, or (b) there is a change in the top five bits between event frames with one or more non‐event frames between them. In such a case, we cannot say with certainty in which frame the change occurred. In case (a), the upper and lower bounds are simply the constant values of $u_{k}=2^{9}\times b_{k}^{\mathrm{MSB}}$ and $l_{k}=2^{9}\times \left(b_{k}^{\mathrm{MSB}}+1\right)$, where $b_{k}^{\mathrm{MSB}}$ denotes the five MSBs of the background value on frame *k*. In case (b), we use the clamping limit to extrapolate upper and lower bounds from the nearest known 14‐bit value (represented by blue dotted lines in Fig. 6).

Step 4. Use the additional information provided by the event intensities to further constrain the bounding curves from Step 3. Note that the change in background level between successive frames depends only on the known 14‐bit event intensity (not to be confused with the complete 14‐bit pixel value). Given a chain of adjacent events for which the 14‐bit background levels could not be exactly reconstructed, we can nevertheless reconstruct the relative 14‐bit values. Only a constant offset is unknown for each such chain of events. For each chain, we determine the minimum and maximum possible offset within the already‐established bounds. The result for each pixel is a pair of bounding curves, as illustrated by the pink shaded region in Fig. 6. In the best case, the curves are identical and the discarded lower nine bits are recovered.

In general, the result of Steps 1–4 applied to a given pixel will be a pair of time series defining the upper (*u*_*k*_) and lower (*l*_*k*_) bounds on the 14‐bit background values for that pixel.

### Observational Uncertainties

Uncertainties on the high‐ and low‐bounding values *u*_*k*_ and *l*_*k*_ are inferred from known detection thresholds used by the onboard RTEPs to identify pixel events. Each pixel is associated with an RTEP that processes the data it produces. For each GLM instrument, a 56 × 32 element table specifies a threshold in raw counts on each of the 56 RTEPs at 32 background intensity levels corresponding to 14‐bit values 0, 512, 1024, … , 15,872. When a pixel is read from the CCD, its background estimate is updated as described by Equation 5. The five MSBs of the current background estimate and an identifying integer for the RTEP are then used to index the table entry containing the appropriate detection threshold. An event is recorded and downlinked if the pixel value exceeds its background estimate by more than the threshold. To construct these tables, billions of data points were collected on orbit and used to analyze the noise characteristics of the system and tune the detection thresholds. The noise was observed to be Gaussian and repeatable across all illumination conditions (Edgington & Tillier, 2016). At each 5‐bit background level on each RTEP, an optimal threshold to noise ratio (TNR) was selected to ensure the return of as much data as possible within the limits of the downlink channel capacity. A TNR represents the number of standard deviations above the mean noise level at which a pixel event is triggered. The TNR for GOES‐16 GLM is currently set to 4.4 near zero radiance, decreasing to 4.0 over bright clouds. For GOES‐17 GLM, the TNR ranges from 4.2 down to 3.9 (C. Tillier, private communication, June 13, 2019). Currently, we have only approximate TNR values with which we construct a 56 × 32 table of pixel noise standard deviations,

(10) $$\sigma \left(i,\;j\right)=\frac{\tau \left(i,\;j\right)}{\mathrm{TNR}\left(i,,,\;,j\right)},$$

where τ(*i*, *j*) denotes the detection threshold at RTEP *i* and intensity *j* and TNR(*i*, *j*) the corresponding TNR.

Note that the characterization of pixel noise described above was based on observations of predominantly background illumination. In using the threshold tables to derive observational uncertainties for impact data, we are assuming this characterization is still adequate. We can justify the assumption in part by observing that light curves of impact events more closely resemble rapidly changing background than the impulses produced by lightning. Spectrally, radiation from an impact within GLM's pass band is a mixture of background‐like continuum radiation and line emissions like those produced by lightning (Fig. 7). Larger impacts will tend to produce higher proportions of continuum radiation (Jenniskens et al., 2018).

**Fig. 7:**
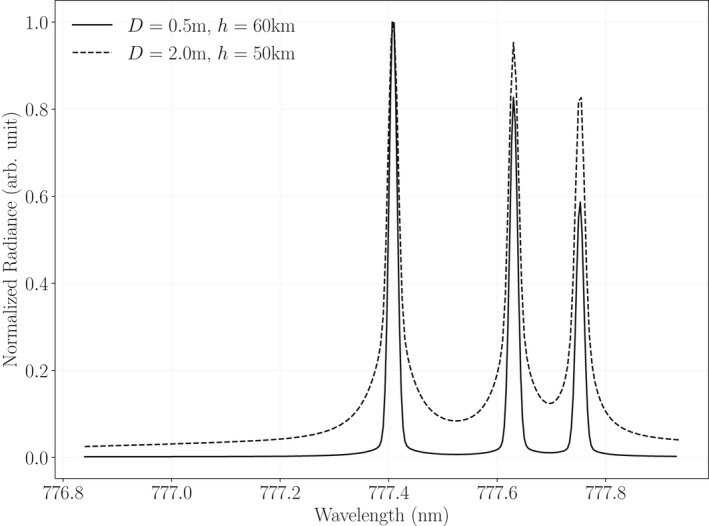
Modeled spectra within the GLM pass band for two hypothetical impactors having different diameters (*D*) and altitudes (*h*), each traveling 20 km s^−1^ upon entry at a 150° view angle (the angle between direction of travel and direction to observer). In general, the greater the impactor's diameter and the lower its altitude when emitting most strongly, the greater the proportion of continuum emission. The total emissions produced by an impact within the GLM pass band can be thought of loosely as a sum of continuum (i.e., blackbody‐like) and line emissions.

Given a 14‐bit value *P* from a pixel processed by RTEP *i*, we interpolate the table σ(*i*, *j*) along row *i* to obtain the observational uncertainty. Because the threshold values are based on empirical measurements, σ accounts for all noise sources affecting the data at the time the thresholds were computed (Edgington & Tillier, 2016). In practice, we usually have only upper and lower bounds on *P*, for which we calculate uncertainties independently with Equation 10. While these values represent the uncertainties of the bounds, the bounds themselves also represent uncertainty in the data and can be treated accordingly by any subsequent analyses.

## CALIBRATION

Pixel event energies in the GLM L2 data product represent the time‐integrated radiant flux within the nominal pass band *before* entering the optics. That is, radiant energy in the solid angle subtended by the pixel without any transmission losses from the optical system. Conversion of the background‐subtracted event intensities (counts) to energy in Joules is part of the L2 ground processing and is accomplished by referencing a lookup table of gain values determined during pre‐flight calibration, as described in the GLM Pre‐Flight Calibration section. Given a pixel event, its location on the CCD and its 5‐bit background value are used to index the appropriate gain factor (J/count), which is multiplied by the intensity to convert it to calibrated energy. Note that a fundamental assumption behind calibrated GLM lightning data is that all lightning emissions are O I line emissions (Jenniskens et al., 2018).

### GLM Pre‐Flight Calibration

Prior to launch, gain tables characterizing the response of each instrument to both static and transient illumination—simulating background and lightning, respectively—were constructed (Edgington et al., 2019; Koshak et al., 2000). The analog‐to‐digital converter (ADC) offsets for each pixel subarray were set to ensure that pixel values were positive at all times in the absence of any illumination. Laboratory temperatures were lower than those expected on‐orbit, adding confidence that pixel values would never drop below zero in flight. The static illuminant (a Labsphere XTH‐2000C integrating sphere) created a flat radiance field with 98% uniformity. Flat field images were collected at 33 evenly spaced intervals over the full dynamic range of the instrument, with dark images collected at each step. A monitoring photodiode was used to record the absolute radiance at each step. Figure 8 shows the results of this procedure within the central 5 × 5 pixel region of each instrument. A gain value for each successive pair of measurements was computed for a total of 32 distinct gain values at each pixel, yielding a 1372 × 1300 × 32 table of values. The table captures nonlinearities in each pixel's response, which are most pronounced near the extremities of the dynamic range.

**Fig. 8:**
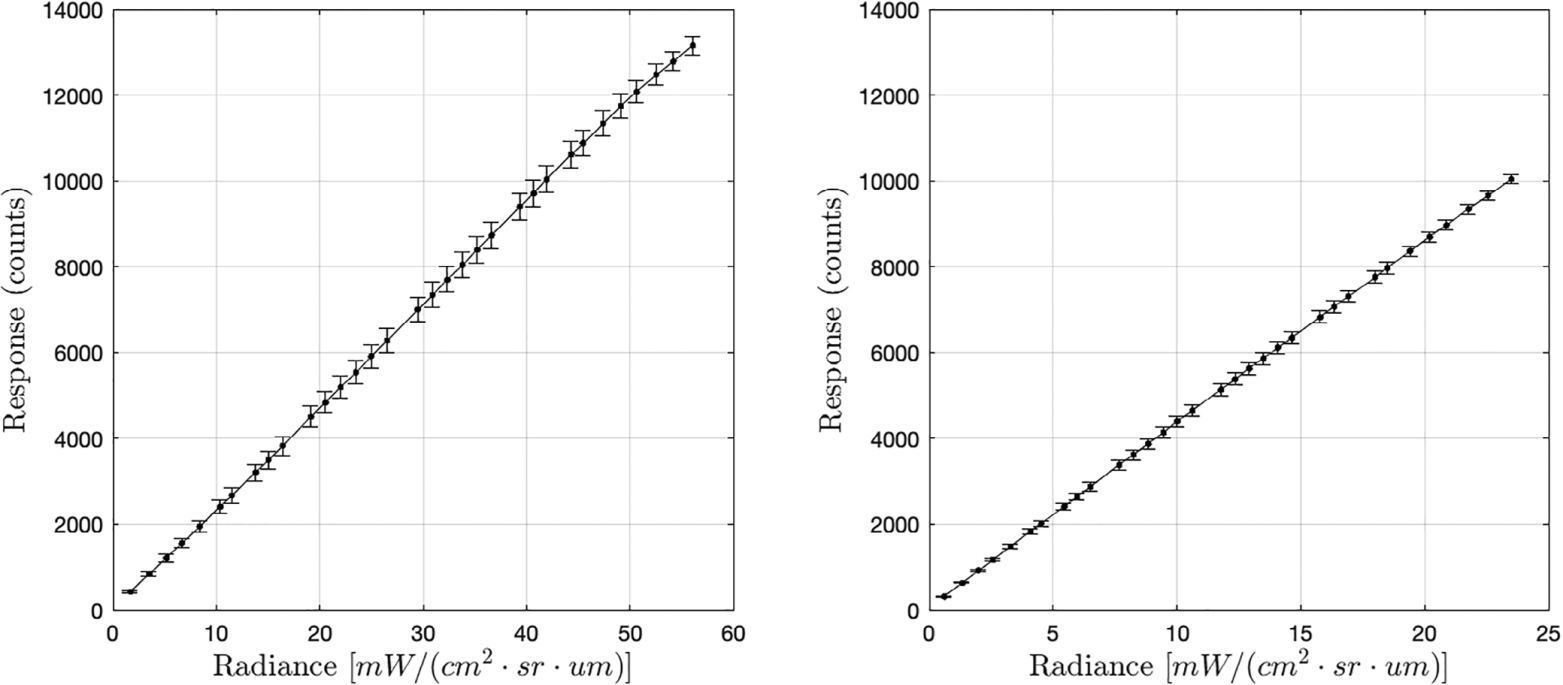
Mean dark‐subtracted responses to steady‐state blackbody‐like illumination in the central 5 × 5 pixel region of GOES 16 GLM (left) and GOES 17 GLM (right). These data were acquired during pre‐flight calibration.

Calibration of the transient response followed the same program with the addition of a pulsing broadband LED into the field of view. Once again, the static flat field was stepped through the entire range of radiances and the integrated energy from the constant‐amplitude LED pulses was varied by stepping the pulse width through two orders of magnitude. The lightning gain table was then produced from the transient response values with additional modeling to account for spectral differences between lightning emissions and the LED.

GLM background and lightning gain tables were delivered in different units as a matter of convenience for their intended uses. For our purposes, we convert the background gain values from radiance units to energy in Joules. From here onward, it is understood that all gain values are expressed in units of J/count.

### Approximating an Impact‐Specific Gain Table

We assume the instantaneous spectrum of an impact's radiant energy within the GLM pass band can be closely approximated by a weighted sum of radiation of two spectral types: blackbody‐like continuum emissions and atomic oxygen *line emissions*. We introduce the concept of a *continuum ratio*, denoted by α, to express the proportions of each spectral type in a given instance. This is simply the ratio of the continuum energy to the total energy within the pass band. The value of α depends mainly on the object's speed, altitude, and ablation behavior (Popova et al., 1998), and also on the view angle between the object's velocity vector and the direction of observation. In some cases, the observed impact emissions will be almost entirely of the continuum variety, and this has been widely assumed in analyses of data from USG satellites (Tagliaferri et al., 1998). Here, we address the general case of calibrating data from impacts emitting arbitrary mixtures of continuum and line radiation.

We also assume the background and lightning gain tables obtained during pre‐flight calibration provide adequate models of each pixel's response to continuum and line emissions, respectively. Note that the differences between background and lightning gain are entirely due to differences in spectral content and not to the transient or static nature of the illumination (C. Tillier, private communication, June 30, 2022). Also note that, taken together, Figs. 8 and 9 imply the response of the GOES 16 GLM to both continuum and line emissions is highly linear. The same is true for the GOES 17 GLM.

**Fig. 9:**
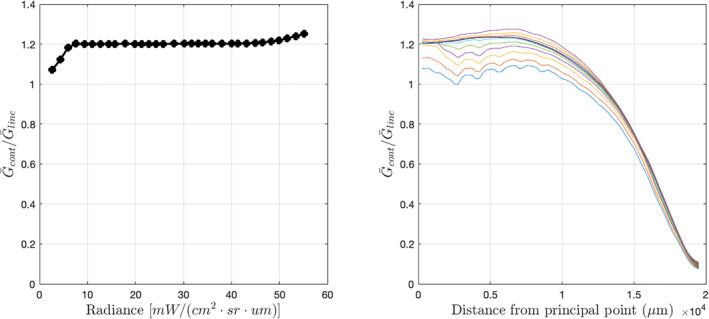
Ratios of GOES 16 GLM background and lightning gain values. Left: mean ratio of background gain to lightning gain in the central 5 × 5 pixel region versus background radiance. Right: mean ratios of background gain to lightning gain within concentric rings around the principal point, each 500 μm wide. Each curve represents mean ratios at one of the 32 background radiance levels. (Color figure can be viewed at wileyonlinelibrary.com.)

The gain tables model variations in pixel response as a function of both illumination intensity and spatial location, with spatial variations depending strongly on spectral content. Within the 1.1 nm wide nominal GLM pass band centered on 777.4 nm, the distribution of spectral energy from continuum emissions can be considered uniform to good approximation. However, the spectrum of O I emissions has three strong line features within the pass band. The system's pass band is sufficiently shifted near the edge of the field of view that it significantly attenuates power from the oxygen triplet, as illustrated by Fig. 10. The gain associated with line emissions will therefore increase near the detector periphery in order to compensate for the increased transmission losses (Fig. 9, right panel), whereas gain values for the continuum component will not.

**Fig. 10:**
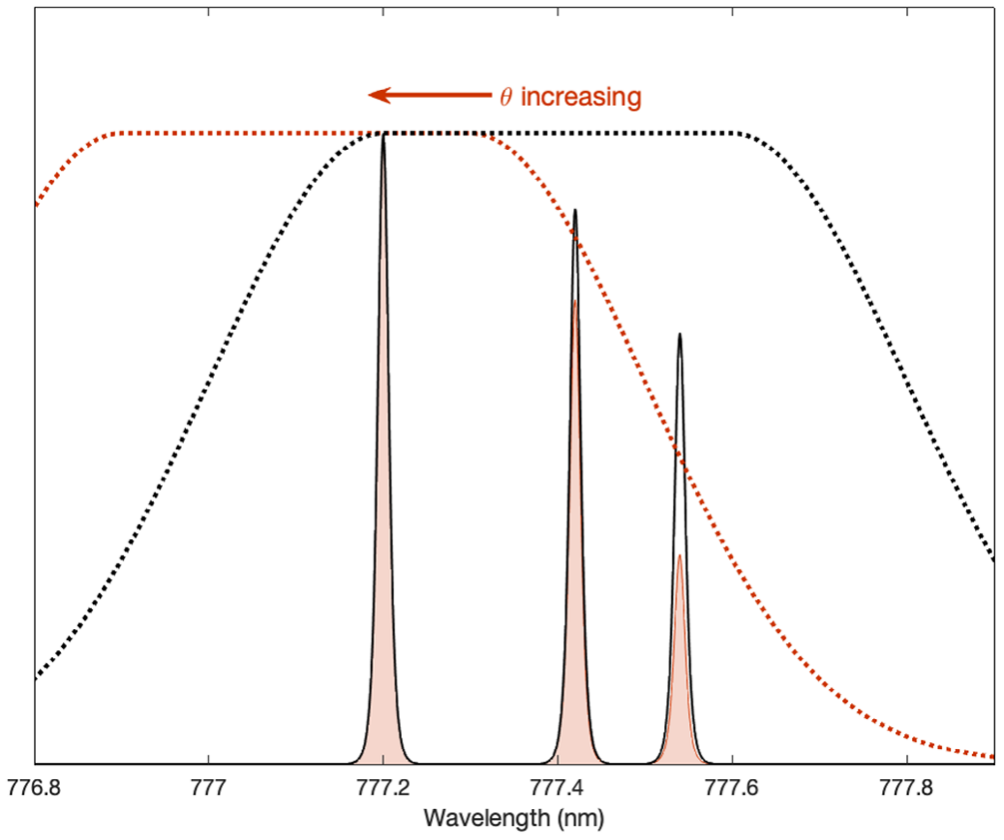
Conceptual illustration of the system's pass band, its dependence on incidence angle (θ), and its interaction with the 777.4 O I oxygen triplet. Solar blocking and rejection filters work in combination with a narrow‐band interference filter to admit power from the triplet. The effective pass band (dotted lines) shifts toward lower wavelengths with increasing incidence angle, which begins to significantly attenuate power at θ > 7° (Jenniskens et al., 2018). (Color figure can be viewed at wileyonlinelibrary.com.)

For each pixel and frame at which a pixel event was recorded, our goal is to determine the radiant energy from the impact within the nominal pass band. The total energy is the sum of the energies from the two spectral components *E*_cont_ = α*G*_cont_(*x*, *y*, *z*)*P*_impact_ and *E*_line_ = (1 − α)*G*_line_(*x*, *y*, *z*)*P*_impact_, where *P*_impact_ denotes the portion of the raw pixel value *P* (counts) that is due to impact radiation and *G*_cont_ and *G*_line_ denote the gain tables for continuum and line emissions, respectively, in units of J/count. Integers *x* and *y* denote the pixel coordinates and *z* an integer in the range [0,31] determined by the five MSBs of *P*, or $\left\lfloor P/512\right\rfloor$. The effective gain for an impact‐triggered pixel event is given by

(11) $$G_{\text{impact}}\left(x,y,z,\alpha \right)=\alpha G_{\text{cont}}\left(x,y,z\right)+\left(1\;-\;\alpha \right)G_{\text{line}}\left(x,y,z\right).$$

Note that, in general, the continuum ratio will depend on a number of time‐dependent factors, including impactor velocity and altitude.

### Calibration with Known Parameters

If both the 14‐bit value *P* and 14‐bit background level *P*_*bg*_ (not to be confused with the onboard background estimate *b* introduced in the 14‐Bit Background Reconstruction and Bounding section) at pixel *x*, *y* are known, and if the continuum ratio α is also known, then the impact‐related portion of the radiant energy in the pixel's field of view (FOV) is given by

(12) $$E_{\text{impact}}=G_{\text{impact}}\left(x,y,\left\lfloor P/512\right\rfloor \right)\left(P\;-\;P_{\mathrm{bg}}\right),$$

and the associated uncertainty by

(13) $$\Delta E_{\text{impact}}=G_{\text{impact}}\left(x,y,\left\lfloor P/512\right\rfloor \right)\sigma ,$$

where σ denotes the observational uncertainty, as calculated in the Observational Uncertainties section. Rather than indexing *G*_impact_ with $\left\lfloor P/512\right\rfloor$, one could also interpolate the table values at *P* for a more precise, though not necessarily more accurate, result.

In most cases, the 2‐min background images in the L0 data provide a basis for high‐quality estimates of the true background levels at each pixel. We describe this approach in further detail in the Results and Discussion section. The question of how these estimates might be improved is left as future work.

### Calibration with Uncertain Parameters

In practice, the 14‐bit pixel value *P* is often not precisely known but can always be bounded. Continuum ratios are also rarely known with much certainty, though by definition, they must lie within the interval [0,1] and it may be possible in a given case to say with some confidence that they lie within a tighter interval. If we relax the assumption that *P* and α are known, we can treat them instead as bounded random variables having unknown distributions. In this case, we apply Equations 12 and 13 to the extreme values of each parameter. This results in four values corresponding to (*P*_max_, α_max_), (*P*_max_, α_min_), (*P*_min_, α_max_), and (*P*_min_, α_min_). Of these, we take the minimum and maximum values, along with their uncertainties, to be the calibrated upper and lower bounds. A more advanced treatment, which we leave as future work, might model *P*_*k*_ and α_*k*_ (with *k* denoting frame number), as Markov random processes.

Because we assume in our analyses that the 14‐bit background is known, the total uncertainty in our model will comprise three components, as illustrated in Fig. 11. The outer edges of region (b) define the bounds on the calibrated values ignoring the intrinsic uncertainties. The innermost shaded region (a) represents the calibrated upper and lower bounds of *P* under an arbitrary value of α (we chose 0.5) and is provided to help visualize the effects of this component on the total uncertainty. Region (c) represents the contribution of intrinsic pixel noise to the total uncertainty and indicates the 3σ confidence interval on the calibrated values. In the legend, Δ*E* denotes the propagated value of σ according to Equation 13.

**Fig. 11:**
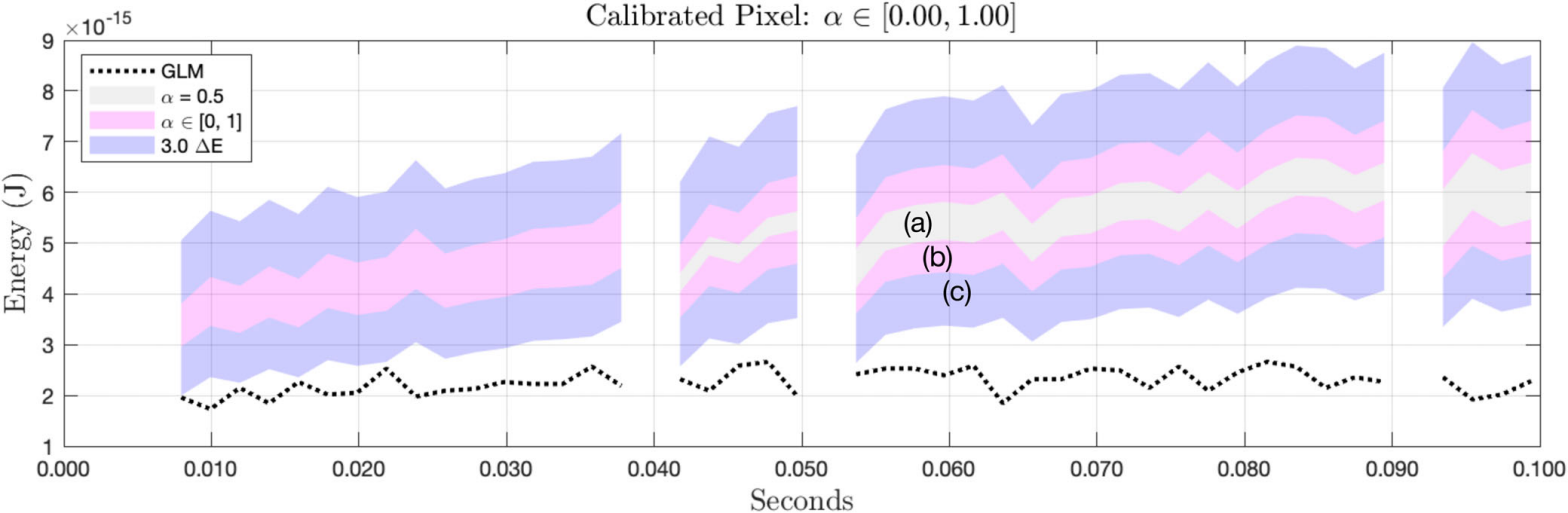
Components of the total uncertainty in the calibrated values. a) Propagated bounds of the 14‐bit pixel value, assuming α = 0.5. Because the leftmost segment of the time series can be reconstructed exactly, there is no uncertainty in the 14‐bit value. b) Widening of bounds due to uncertainty in α. c) The three standard deviation contour of the Gaussian pixel noise. The extremes of the pink region represent the bounds on energy values in the absence of pixel noise, while the outer extremes of the blue region represent the 99.7% confidence interval including pixel noise. (Color figure can be viewed at wileyonlinelibrary.com.)

## Results and Discussion

The calibration procedure described in the Calibration section relies on the availability of high‐quality estimates of the background level *P*_*bg*_ at each pixel. While background levels can be estimated any number of ways, a simple and reasonable approach is to interpolate the 2‐min, 14‐bit background images in the L0 data files. This approach should deliver accurate estimates provided that (1) the background level in the region of interest is slow‐varying and (2) the time interval in which the impact occurs does not overlap any of the background images used. Because background levels have been observed to change rapidly in response to solar glint, and because there may be other phenomena that can cause similar effects, the first assumption may not always be valid. A potential solution is to build a daily map of background variation as a function of time and flag regions that are highly variable on short time scales. The second assumption can be checked during processing and background images that overlap the impact can be omitted.

It might seem reasonable to assume that the correct background value should closely match the onboard GLM estimate on the first recorded event at each pixel. For large and fast‐moving impactors at steep angles of entry, this is probably not a bad assumption, but in many cases, there will be a slow–steady rise in brightness before GLM begins recording events. In such cases, we would expect our background estimate to be a bit lower than the onboard estimate for the first recorded event. Our energy estimates will tend to be a bit higher as a result, which is what we see in most cases.

As noted in the Approximating an Impact‐Specific Gain Table section, the proportions of observed continuum and O I line emissions from a given impact depend on the impactor's velocity, altitude, ablation behavior, and the observer's view angle. Modeling the relationship between the continuum ratio α and such impact parameters is the subject of ongoing work. For now, we make no assumptions about the value of α, except that it lies on the interval [0,1].

The right‐hand plot of Fig. 9 implies that the sensitivity of the calibrated output to the continuum ratio increases dramatically near the edges of the field of view. We can most easily see this effect in the calibrated results by examining calibrated impact observations both near the center of the FOV (Fig. 12) and on the periphery (Fig. 13). Note that in the upper right‐hand plots of each figure, there are points labeled *MSB transition*. These are points at which there was a change in the returned 5‐bit background value. At these points, the binary 14‐bit value can be exactly reconstructed by simply appending nine zeros to the 5‐bit value. We can then infer chains of adjacent values exactly by applying Equations 9 and 7.

**Fig. 12:**
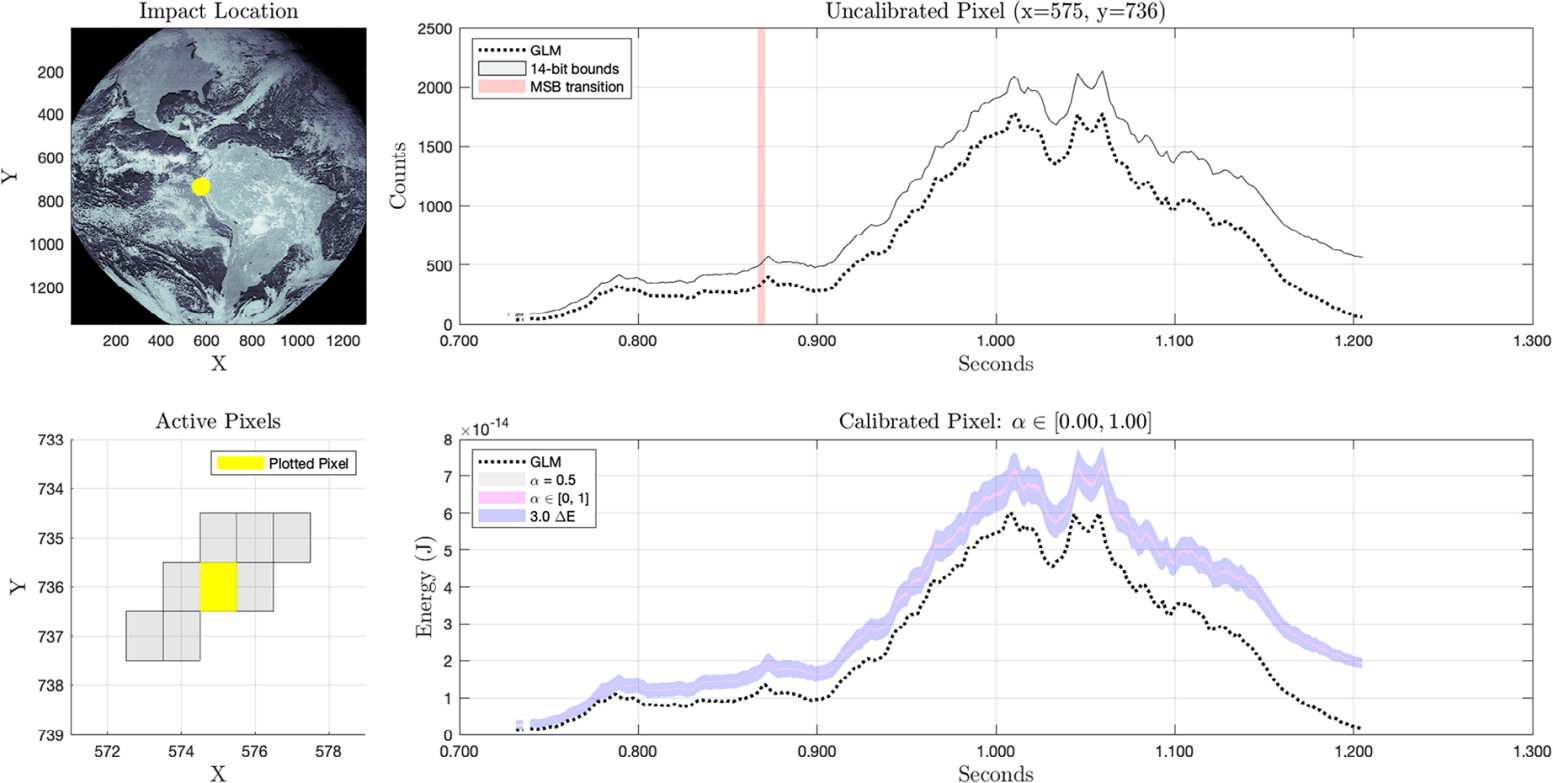
Results for a bolide impact imaged by the central region GOES 16 GLM detector. The impact occurred over Ecuador at 3.65°S, 80.59°W on July 20, 2020 at 10:30 UTC. The pixel shown recorded the most energy of eight pixels active during the impact. In this region and at these energies, the calibrated result is largely insensitive to the value of α. Uncertainty in the calibrated pixel time series (lower right) is dominated by the intrinsic pixel noise (blue). (Color figure can be viewed at wileyonlinelibrary.com.)

**Fig. 13:**
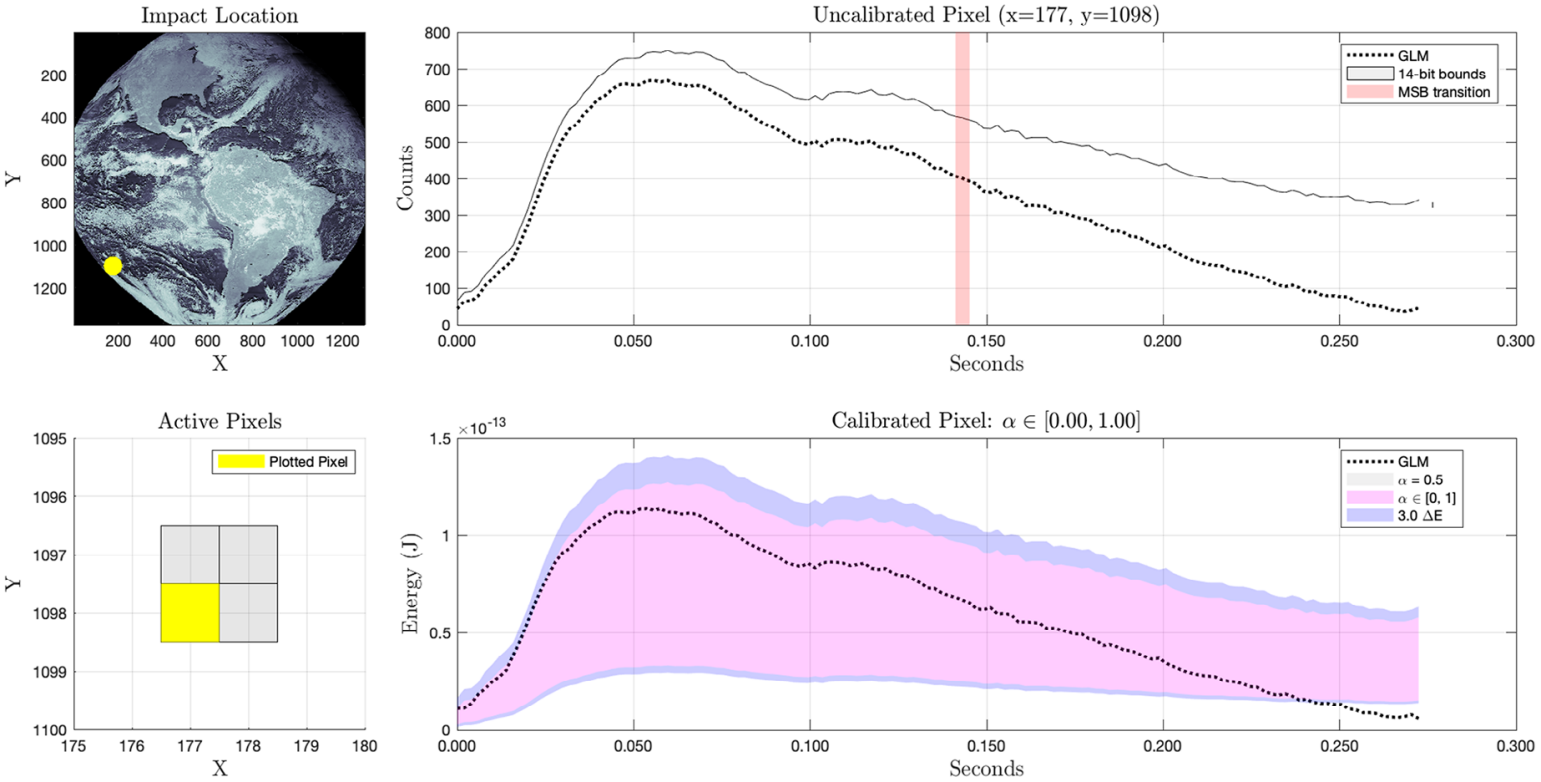
Results from an impact imaged near the periphery of the GOES 16 GLM detector. The impact occurred at 33.4°S, 122.1°W on July 20, 2020 at 11:21 UTC. The pixel shown recorded the most energy of four pixels active during the impact. In this region, the calibrated result is highly sensitive to the value of α and uncertainty in its value dominates the total uncertainty in calibrated values. The upper edge of the pink region corresponds to α = 0 and the lower edge to α = 1.0. Unless the true value is near zero, the energy reported in the GLM L2 product has been overestimated at the beginning of the time series and underestimated toward the end. (Color figure can be viewed at wileyonlinelibrary.com.)

Figure 14 shows the calibrated results of our procedure on data from an April 13, 2021 impact near Grand Bahama and compares them to the reported energies delivered by GLM L2 ground processing. Events were recorded on two GLM pixels. The pixel recording higher amplitudes also recorded a single contiguous chain of events, allowing for perfect reconstruction of 14‐bit values. The reconstructed pixel shows between 19% and 23% more observed energy than was reported in the L2 data product. The lower amplitude pixel recorded two contiguous chains of events, neither of which contained a change in the ninth bit of the onboard background estimate. This means the 14‐bit background was not exactly known at any point on this pixel and could only be bounded. Therefore, assuming our re‐estimated background is correct, over 28% more radiant energy from the impact (total on both pixels) was observed than was reported in the L2 data product. Our background estimate was taken directly from the calibrated background image acquired within 2 min of the impact, under the assumption that the local background illumination was stable before and after.

**Fig. 14:**
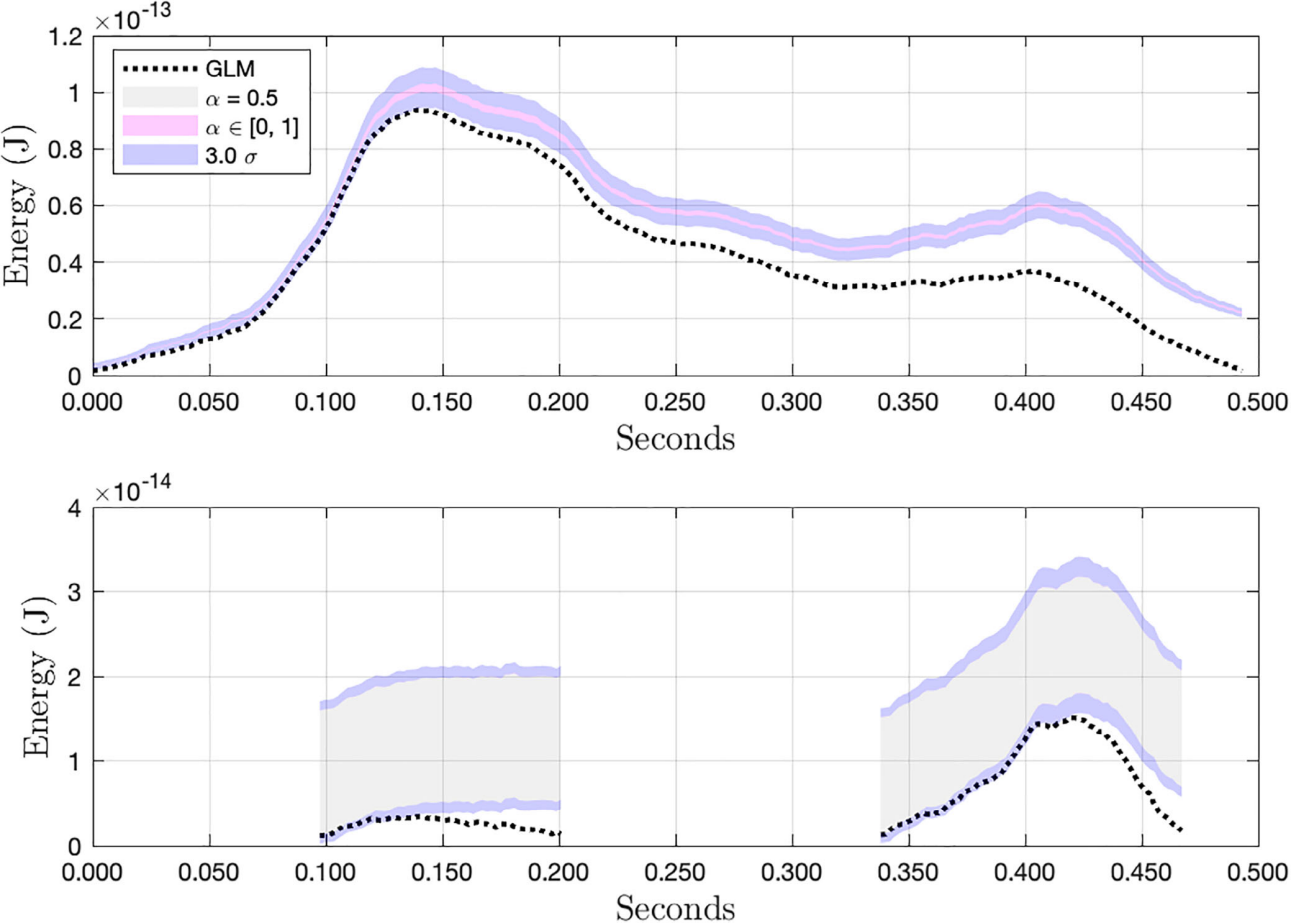
Comparison of calibrated and background‐subtracted pixel time series from a bolide detected at 26.95°N, 79.11°W on April 13, 2021 at 2:16 UTC. At least 28% more radiant energy from the impact was observed than was reported in the L2 data product. (Color figure can be viewed at wileyonlinelibrary.com.)

The Grand Bahama bolide was the subject of in‐depth analysis by a team at the Florida Institute of Technology (Hughes et al., 2022). They derived their light curve primarily from GLM L2 data. Camera 20A from NASA's All Sky Fireball Network captured supplementary data points not represented in the GLM data, but the additional luminous energy was deemed negligible. A blackbody model was then anchored to the GLM energies to extend the energy estimates to the full visual range. It would be interesting to explore how corrections to the calibrated GLM energies indicated by our results might affect the results of their analyses. Their analysis of data from the NASA 20A camera also shows that the bolide was visible nearly 3 s before GLM began recording events, so the onboard background estimates would have been rising in response to it well before the first recorded pixel events. This illustrates the mechanism, described earlier, by which GLM event energies may be underestimated even at the time of the first recorded event.

Figure 15 shows the calibrated result for another pixel from the July 20, 2020 bolide over Ecuador introduced in Fig. 12. This pixel is the third most energetic of eight pixels that were active (i.e., recording events) during the impact. Exact reconstruction of 14‐bit values was possible for the chain of pixel events on the left, but not for the chain on the right. While the GLM L2 data product shows a decreasing trend in energies from about 0.44 s onward, our result indicates a second peak in the recorded energy. If we examine the bounds of our calibrated results for all eight pixels comprising the observations of this impact, we find that the total energy observed was between 54% and 78% greater than the energy reported in the L2 data product. Again, the accuracy of this result depends on the accuracy of our background estimate.

**Fig. 15:**
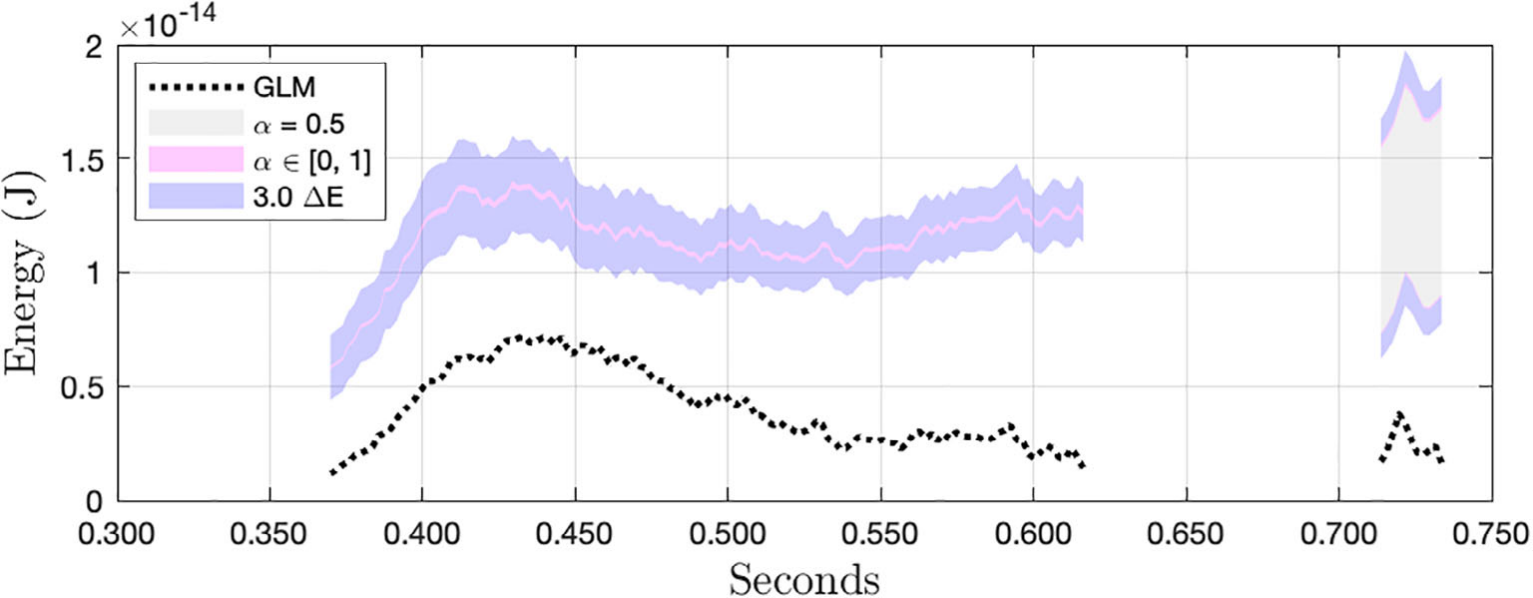
The third‐most energetic pixel from the July 20, 2020 bolide introduced in Fig. 12. This pixel is of interest because, while the GLM L2 data product shows decreasing flux, the corrected result appears to show an energy peak. (Color figure can be viewed at wileyonlinelibrary.com.)

Aside from the differences in reported energy, the restoration of misclassified background flux potentially changes one's interpretation of qualitative features in the data. In both Figs. 14 and 15, what looks like periods of nearly constant brightness in the L2 data more closely resemble flares in our results.

## Conclusions

The problem of using GLM data to accurately estimate light curves for impact events naturally breaks down into two steps: (1) correcting errors in the energies derived from the downlinked pixel observations of the impact and (2) effectively or explicitly filling in missing observations to produce a light curve for the impact event as a whole. This paper has addressed the first step, while the second is the subject of ongoing work.

We have described methods for clustering impact‐related GLM pixel events, aligning pixel‐level data in the L0 and L2 data products, reconstructing or bounding the 14‐bit onboard pixel values, and calibrating the results. Sources of uncertainty were identified along with our approaches to managing them. These sources included intrinsic noise, information loss due to onboard processing, and uncertainty in the spectral content of the radiant energy from an impact event. A key assumption we have made is that these three sources dominate the total uncertainty in the results.

We leave it to future work to account for additional sources, such as uncertainty in background estimates or in the results of the overshoot correction, should they prove to be significant. We are currently looking more closely at whether the overshoot algorithm discussed in the GLM False Event Filters section is appropriate for impact data, and can disable it if necessary. Work on modeling the relationship between impact parameters and spectral content is ongoing. We also leave to future work the application of Markov processes models to the 14‐bit pixel values, continuum ratios, and potentially other uncertain quantities (e.g., background) expected to vary smoothly with time.

Under our stated assumptions, we have shown how to bound the radiant energy measured by a given pixel with high confidence. By comparison with our results, we have given examples of under‐ and over‐reporting of observed radiant energies for bolides in the GLM lightning data product. Under‐reporting is primarily the result of GLM's onboard processing algorithms misclassifying impact energy as background. In addition to introducing quantitative errors in the reported energies, this behavior has also been shown (Figs. 14 and 15) to obfuscate broad peaks in the pixel time series, possibly changing the qualitative interpretation of the data in terms of flares, fragmentations, etc. Over‐reporting occurs mainly near the edges of the detector as a result of the GLM ground processing applying lightning‐specific corrections to impact observations. We have shown how to correct this effect if the proportions of continuum and line emissions from the impact event are approximately known.

## Data Availability

The data that support the findings of this study are available from the corresponding author upon reasonable request.
